# Consumption of 100% Juice and Diluted 100% Juice Is Associated with Better Compliance with Dietary Guidelines for Americans: Analyses of NHANES 2017–2023

**DOI:** 10.3390/nu17162715

**Published:** 2025-08-21

**Authors:** Rozenn Gazan, Matthieu Maillot, Adam Drewnowski

**Affiliations:** 1 MS-Nutrition, 13005 Marseille, France; rozenn.gazan@ms-nutrition.com (R.G.); matthieu.maillot@ms-nutrition.com (M.M.); 2 Center for Public Health Nutrition, University of Washington, Seattle, WA 98195, USA

**Keywords:** fruit juice, diluted fruit juice, Dietary Guidelines for Americans, American Academy of Pediatrics, Healthy Eating Index 2020, Nutrient Rich Food Index NRF9.3

## Abstract

**Background**: The Dietary Guidelines for Americans (DGA) and the American Academy of Pediatrics recommend limiting 100% juice consumption to 0.5–1.25 cups/day and to no more than one half of total fruit intake. **Objective**: To explore the dietary benefits of consuming 100% fruit juice and diluted 100% juice across diverse socio-demographic strata in the US. **Methods**: Consumption patterns for 100% juice and diluted 100% juice were examined by sex, age group, income-to-poverty ratio (IPR), and race/ethnicity. Dietary intakes came from the National Health and Nutrition Examination Survey (NHANES 2017–2020 and 2021–2023). The Healthy Eating Index 2020 (HEI 2020) and diet-level Nutrient Rich Food (NRF9.3) scores were the two measures of diet quality. The amounts of 100% juice consumed were compared to published DGA recommendations. **Results**: The consumption of 100% juice was greatly below that of water, milk, and sugar-sweetened beverages (SSBs). The consumption of diluted 100% juice was very low. Consumers of 100% juice had higher HEI 2020 scores (53 vs. 48) and diets with less added sugar and more total fruit, more potassium, calcium, and vitamin C. About 88% of the NHANES sample consumed <4 oz/day (1/2 cup) of 100% juice and most derived at least 50% of fruit from whole fruit, though some variation by income and race/ethnicity was observed. About 93% of the sample consumed <1 cup/day (8 oz) of 100% juice. Lower income groups consumed less whole fruit and more 100% juice. **Conclusion**: The consumption of 100% fruit juice was a marker of healthier dietary choices. The observed social gradient suggests that 100% fruit juice may provide valuable nutrients to populations who may be unable to afford or access whole fruit. **Public health recommendations**: The consumption of 100% fruit juice by some population subgroups could be increased. Fruit juice was not displacing whole fruit, and current consumption was well below the current DGA recommended values.

## 1. Introduction

The Dietary Guidelines for Americans 2020-2025 (DGA) recommend reducing the consumption of 100% juice on the grounds that juice “can contribute extra calories when consumed in excess” [1,2]. These limitations apply to pasteurized 100% juice and to 100% juice diluted with water and without added sugars [1,2]. The specific DGA amounts for 100% fruit juice differ by age, ranging from ^1^/_2_ cup (4 oz) for children aged 2–3 y to 1.25 cups (10 oz) for men aged ≥19 y. To insure adequate fiber intakes, the DGA recommends that at least half of the total fruit should come from whole fruit.

According to the DGA, up to ^1^/_2_ cup of 100% juice can fit in a healthy dietary pattern. Yet the consumption of 100% fruit juices in the US is low and 100% fruit juices provide relatively few calories [3]. Studies have shown that sugar-sweetened beverages (SSBs) are consumed far more often and in much greater amounts, especially by older children and by young adults [4]. Even so, it is the consumption of 100% fruit juice that has been associated with an increased risk of childhood obesity [5,6,7,8].

The decline in consumption of SSBs and other beverages [9,10,11] has been well-described. By contrast, fewer US-based studies have explored current consumption patterns for 100% juice and diluted 100% juice [12]. Orange juice and apple juice are the two principal 100% fruit juices in the US diet [5]. Past studies have shown that citrus juices were an important source of bioactive flavanols [13,14], particularly among children. In past studies, also based on NHANES data, beverage patterns that were primarily composed of milk and 100% juice were associated with higher-quality diets overall as compared to beverage patterns that were primarily composed of sugar-sweetened beverages [15].

The present analyses used dietary intake data for children, adolescents, and adults from two cycles of the National Health and Nutrition Examination Survey (NHANES 2017–2020 and 2021–2023). Consumption patterns for 100% juice and diluted 100% juice were examined across key population subgroups defined by socioeconomic status and race/ethnicity. The hypothesis was that the consumption of 100% juice and diluted 100% juice would be associated with higher diet quality and with lessened disparities in access to healthy diets. The amounts of 100% juice consumed were further compared to standards proposed by the DGA and the American Academy of Pediatrics [16].

## 2. Materials and Methods

### 2.1. Population Data and Surveys

Dietary intake data of 4086 individuals aged 5–19 years and 10,925 adults came from two most recent cycles of the National Health and Nutrition Examination Survey (2017–2020 and 2021–2023). The NHANES 24 h recall uses a multi-pass method, asking for the types and amounts of all foods and beverages consumed in the prior 24 h, from midnight to midnight [17]. This procedure is conducted by a trained interviewer using a computerized interface. The recall first identifies a quick list of foods and beverages consumed. The time and occasion for each food item are also obtained. A detailed cycle then records the amounts consumed, followed by a final probe for potentially forgotten foods (beverages, condiments). Dietary intakes are provided for 24 h and for each eating occasion. This study was based on 2 days of dietary recall.

The Food Patterns Equivalents Database (FPED) [18] converts each food into the number of cups of food groups that are listed in the Dietary Guidelines for Americans. For instance, in FPED, 100 g of 100% fruit juice beverage equals a 0.4-cup equivalent of fruit juices (1 cup equals around 240 g of juice). FPED was available only for NHANES cycle 2017–2020; therefore, 29 foods or beverages consumed only in the 2021–2023 NHANES cycle were linked to an existing food in the FPED file.

### 2.2. Participant Characteristics

To ensure representativeness, NHANES used multistage probability sampling. Primary sampling units (PSUs) were selected with probability proportional to size. Then, segments were selected within the PSUs and households randomly selected within segments. Certain groups were oversampled (e.g., older adults and lower income groups) to improve the precision of estimates for those groups. For the present analyses, NHANES participants were stratified by sex (male, female), age, race/ethnicity, and the family income-to-poverty ratio (IPR) level. The IPR is the ratio of the total annual household income before taxes to the US Census Bureau poverty threshold for the survey year. Those calculations were performed by the National Center for Health Statistics. Age groups for children were young children (5–8 y), older children (9–13 y), and adolescents (14–19 y). Age groups for adults were 20–30 y, 31–50 y, 51–70 y, and >70 y. The cut-points for the family income-to-poverty ratio (IPR) were as follows: <1; 1–1.99; 2–3.49; and ≥3.5. Demographic NHANES questionnaires provided data on race/ethnicity, defined as non-Hispanic white; non-Hispanic black, Mexican American, other Hispanic, and other/mixed race.

### 2.3. Classification of Beverages

Water and beverages were classified based on the What We Eat in America (WWEIA) Food Categories and data from FPED (Table 1). Beverage categories were as follows: (1) 100% fruit juices; (2) 100% diluted fruit juices without added sugar; (3) milk and flavored milk; (4) drinking water, tap and bottled; (5) other caloric sugar-sweetened beverages with ≥50 kcal/240 g; (6) other non-caloric and low-calorie beverages (<50 kcal/240 g).

### 2.4. Healthy Eating Index 2020 and Nutrient Rich Food Index

The Healthy Eating Index (HEI 2020) is a 100-point scale, composed of 13 sub scores that include total and whole fruit, vegetables, dairy, whole grains, and protein foods as well as added sugar, sodium, refined grains, and saturated fat. The HEI serves as a measure of compliance with the Dietary Guidelines for Americans [19].

The diet-level Nutrient-Rich Food Index (NRF9.3) is composed of two sub scores, NR9 and LIM. The positive NR9 sub score is the sum of percent daily values for 9 nutrients to encourage: protein, fiber, calcium, iron, potassium, magnesium, vitamin A, vitamin C, and vitamin D. The negative LIM sub score is based on the sum of excess percent daily values for 3 nutrients to limit: saturated fat, added sugar and sodium. Each daily nutrient intake was adjusted for 2000 kcal. Nutrient standards were taken from the Food and Drug Administration values for a 2000 kcal/day diet and are shown in Table 2.

### 2.5. Analysis

The NHANES sample was first stratified by sex, age group, income-to-poverty ratio (IPR), and race/ethnicity. Associations between the percentages of consumers of 100% fruit juices and age groups or socio-demographic variables were assessed by performing Chi-square tests. Amounts of beverages consumed in g/day were expressed as means and standard deviations, 95% confidence limits, and as percentile distributions (10th, 25th, 50th, 75th, 90th percentiles). Analyses of beverage consumption were conducted for the whole population, and comparisons were made by age group, sex, IPR, and race/ethnicity using generalized linear model.

Statistical analyses were conducted for 100% and diluted juices. Consumers and non-consumers were compared based on mean dietary quality scores (HEI and NRF9.3) adjusting for socio-demographic variables and total energy intakes using a generalized linear model.

Compliance with the DGA was assessed by estimating the proportion of individuals consuming <0.5 cups of 100% juice, 0.5 to 1 cup, 1 to 1.5 cup, 1.5 to 2 cups, and ≥2 cups. These proportions were calculated for the whole population, by age group, sex, IPR, and race/ethnicity.

Amounts of 100% juice were further compared to the amounts of whole fruits consumed. Those NHANES participants who consumed any whole fruits were identified. The fruit categories were as follows: other fruit and fruit salads; bananas; apples; pears; dried fruits; blueberries and other berries; grapes; strawberries; citrus fruits; pineapple; mango and papaya; melons; peaches and nectarines, and baby fruit. Analyses calculated the ratios between the consumption of 100% fruit juices and whole fruits in cup equivalents. Mean ratios were estimated for the whole population and age group, sex, IPR, and race/ethnicity using generalized linear models. All analyses used weighting factors to account for the complex survey design of NHANES data and are representative of the US population. Data analyses used R software 4.4.2.

## 3. Results

### 3.1. Characteristics of the NHANES Sample

Table 3 shows socio-demographic characteristics of the NHANES sample by age group, sex, the family income-to-poverty ratio, and race/ethnicity. The sample was composed of children aged <19 y (n = 4086) and adults aged >19 y (n = 10,925). The variables of interest were age group, sex, IPR, and race/ethnicity.

### 3.2. Consumers and Non-Consumers of 100% Juice and Diluted 100% Juice

Only 24.5% of the NHANES sample consumed any 100% fruit juice or diluted 100% fruit juice on 2 days of NHANES (Table 4). Higher consumption prevalence was associated with younger (<13 y) and older (>70 y) age groups (*p* < 0.001) and with lower IPR (*p* < 0.001). Non-Hispanic White groups were less likely to consume 100% juice than Non-Hispanic Black, other Hispanic, and Mexican American groups (*p* < 0.001). No associations by sex were observed.

### 3.3. Distribution of Beverage Consumption by Age Group

**Figure 1:**
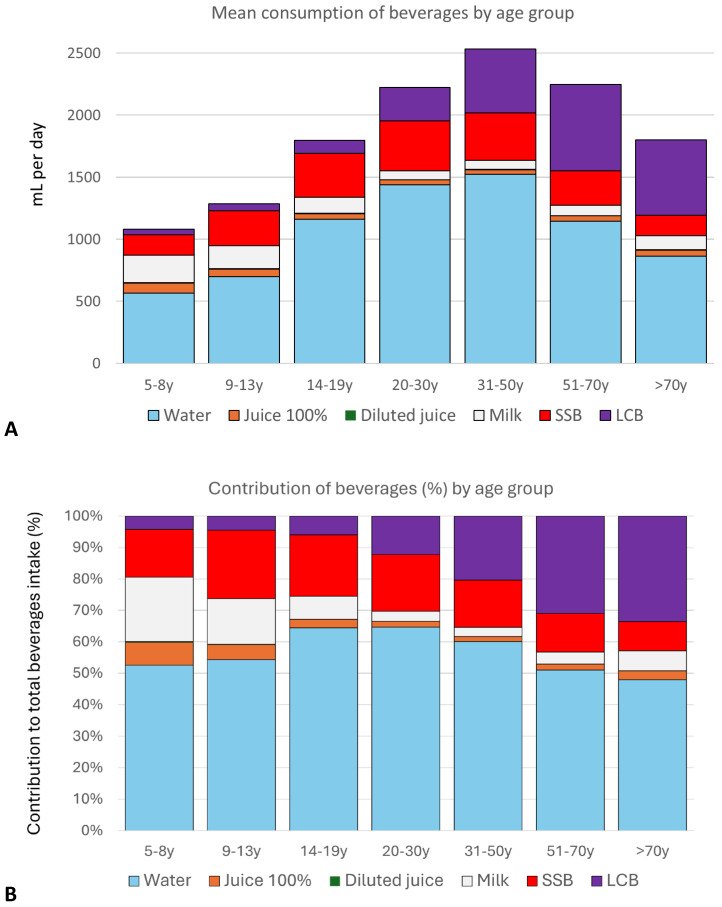
Mean daily consumption of beverage categories in grams (**A**) and as a percentage of total mean beverage consumption (**B**) by age group.

The distribution of 100% fruit juice (+diluted) consumption by age group and beverage category is shown in Figure 1. Means in absolute mean amounts (in g/day) are shown in Figure 1A and means in relative percentages are shown in Figure 1B. The most consumed beverage was water, bottled and tap. Milk and flavored milk consumption was highest among children aged 5–8 y and declined with age with a small increase observed after the age of 70 y. The consumption of regular sugar-sweetened beverages was highest among older children (9–13 y) and adolescents (14–19 y) and declined with age. The consumption of low-calorie beverages increased with age, reaching maximum values after age 51 y. Fruit juices 100% (and diluted, whether considered independently or as a sum) were consumed by younger (ages 5–8 y) and older children (ages 9–13 y), with amounts declining with age (except for >70 y).

### 3.4. Distribution of Beverage Consumption by Socio-Demographics

**Figure 2:**
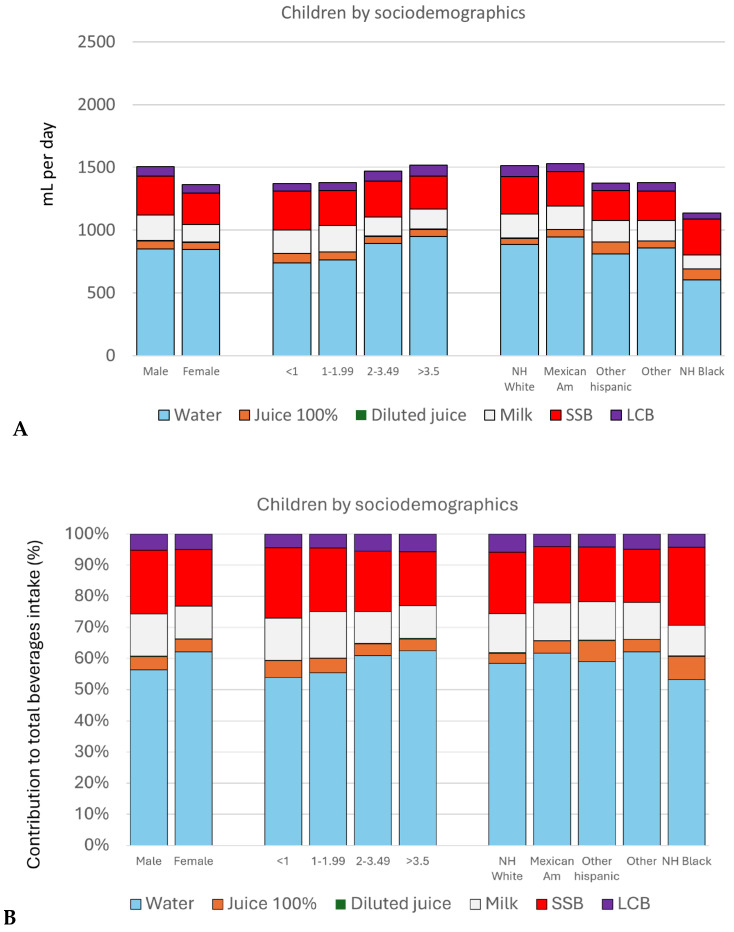
Mean consumption of beverages by category in g/day (**A**) and as a percent of total mean beverage consumption (**B**) among children (ages ≤ 19 y) by socio-demographics. SSB, sugar-sweetened beverages; LCB, low-caloric beverages;  *p* values for comparisons by demographics were as follows: for 100% diluted fruit juice and IPR, *p* = 0.004; for milk and flavored milk and race/ethnicity, gender and IPR, p = 0.019, <0.001, and <0.001, respectively; drinking water and race/ethnicity and IPR, *p* = 0.018 and 0.005, respectively; and between SSB consumption and gender, *p* < 0.001.

Figure 2 shows beverage consumption by category among children (ages < 19 y) by socio-demographics. Mean amounts (g/day) are shown in Figure 2A, and relative percentages are shown in Figure 2B. Supplementary Table S1 shows detailed distributions of daily consumptions (g/d) by age classes. There were no differences between the sexes for plain water (tap and bottled), 100% fruit juices, and low-calorie beverages. Higher consumption of water was associated with higher IPR. Higher consumption of milk and flavored milk was associated with lower IPR. Among children, there was no significant relation between household IPR and the consumption of 100% juices, sugar-sweetened beverages, and low-calorie beverages. Rather, an association between 100% diluted juices and higher IPR was observed.

Consumption patterns for water, milk, and flavored milk varied by race/ethnicity. Mexican American children drank the most water; NH Black children drank the least water. NH Hispanic White and Mexican American drank the most milk, whereas NH Black drank the least milk. Other relationships failed to reach statistical significance.

**Figure 3:**
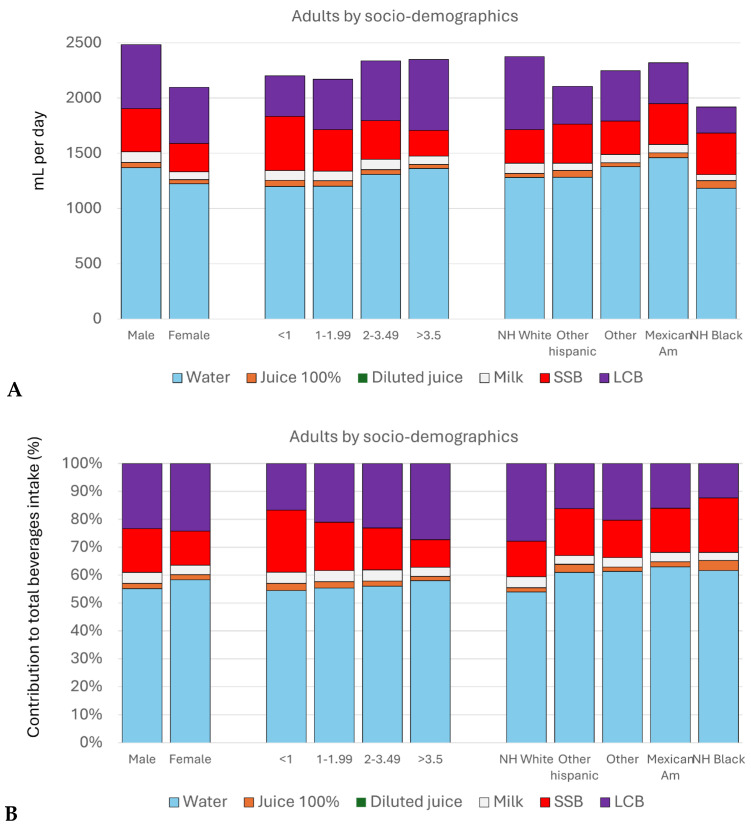
Mean consumption of beverages by category in g/day (**A**) and as a percent of total mean beverage consumption (**B**) among adults (ages >19 y) by socio-demographics. SSB, sugar-sweetened beverages; LCB, low-caloric beverages; *p* values for comparisons by demographics were as follows: for sex and IPR *p* = 0.008 and 0.001, respectively; for milk/flavored milk and sex and IPR, *p* < 0.001 and 0.019, respectively; for drinking water and sex and IPR, *p* = 0.001 and 0.005, respectively; for SSB and sex and IPR, *p* = <0.001; and between LCB and sex and IPR, *p* = 0.001 and <0.001, respectively.

Figure 3 shows beverage consumption by category among adults (ages > 19 y) by socio-demographics. Mean amounts (g/day) are shown in Figure 3A, and relative percentages are shown in Figure 3B. Supplementary Tables S2 and S3 show the distribution of beverage consumption by socio-demographic group among children and adults, respectively. Males consumed more plain water (tap and bottled), 100% fruit juices, milk beverages, sugar-sweetened beverages (SSBs), and low-calorie beverages (LCBs) than did females. Higher consumption of drinking water and LCB was associated with higher IPR. Higher consumption of milk beverages, 100% juices, and sugar-sweetened beverages was associated with lower IPR. The consumption of any beverage did not vary significantly by race/ethnicity.

### 3.5. Compliance with the 100% Juice/Whole Fruit Ratio in the DGA

The present metric of the ratio of 100% juice consumption to whole fruit consumption was restricted to those individuals who consumed whole fruits. The mean ratio was 0.4, meaning that 100% fruit juice accounted for only 0.4 of the total, which is well in compliance with recommended values. A ratio of 0.6 was obtained only for younger children aged 5–8 y.

**Figure 4:**
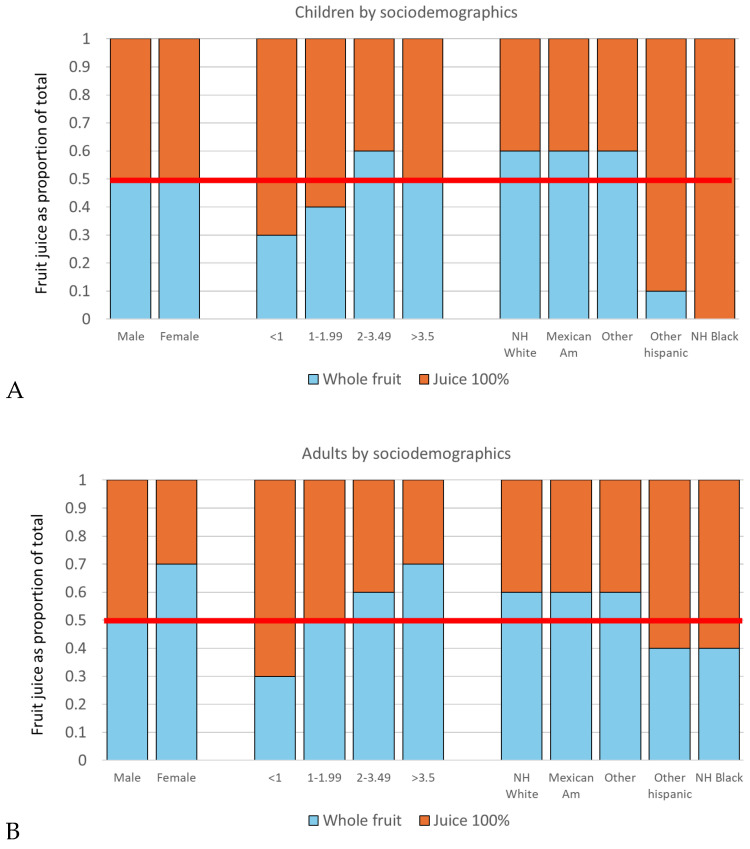
Mean ratios of 100% fruit juices (+diluted) relative to whole fruit by age groups. Data are for consumers of whole fruit (n = 9050) and are for (**A**) children (n = 2447) and (**B**) adults (n = 6603). Difference of ratio between 100% fruit juices and whole fruits were significant for sex for the whole sample (*p* = 0.016) or among adults (*p* = 0.027) and for IPR for the whole sample (*p* = 0.004), children (*p* = 0.01), and adults (*p* = 0.044). Red line indicates an equivalent amount of fruit juice (+diluted) and whole fruit.

Figure 4 shows a significant effect of IPR on 100% juice to whole fruit ratios. Among all individuals, the mean ratios for male and female, 0.5 and 0.4 respectively, were consistent with recommendation of 0.5 or less. A strong social gradient was observed. The proportion of whole fruit was highest for the highest income group (0.7) and lowest for the lowest income group (0.3). Lower IPR was associated with less whole fruit and more 100% fruit juice for all age groups.

The relation between the juice-to-whole fruit ratio and race/ethnicity was not statistically significant (among all individuals or by age groups); however, the proportion of whole fruit was highest for non-Hispanic Whites. In other words, the suggested standard (0.5) was readily met by non-Hispanic Whites and by higher income groups but was not met by lower income and non-Hispanic Black groups.

Table 5 shows that the DGA was followed by 87.1% of the population. About 7.7% consumed between 0.5 and 1.0 cups; 3.1% consumed between 1.0 and 1.5 cups; 0.9% consumed 1.5 to 2.0 cups, and 1.2% consumed 2.0 cups or more. These recommendations were met by 77.0% of children aged 5–8 y and by 82% of children aged 9–13 y. The percent of children consuming more than 1 cup of 100% fruit juice was small: 8.2% for the 5–8 y age group and 6.6% for the 9–13 y age group.

Among all individuals, there were significant effects of IPR, gender, and race/ethnicity (Supplementary Table S4). Separate analyses were conducted for children (<19 y) and for adults (>19 y).

### 3.6. Diet Quality of Consumers and Non-Consumers of 100% Juice

**Figure 5:**
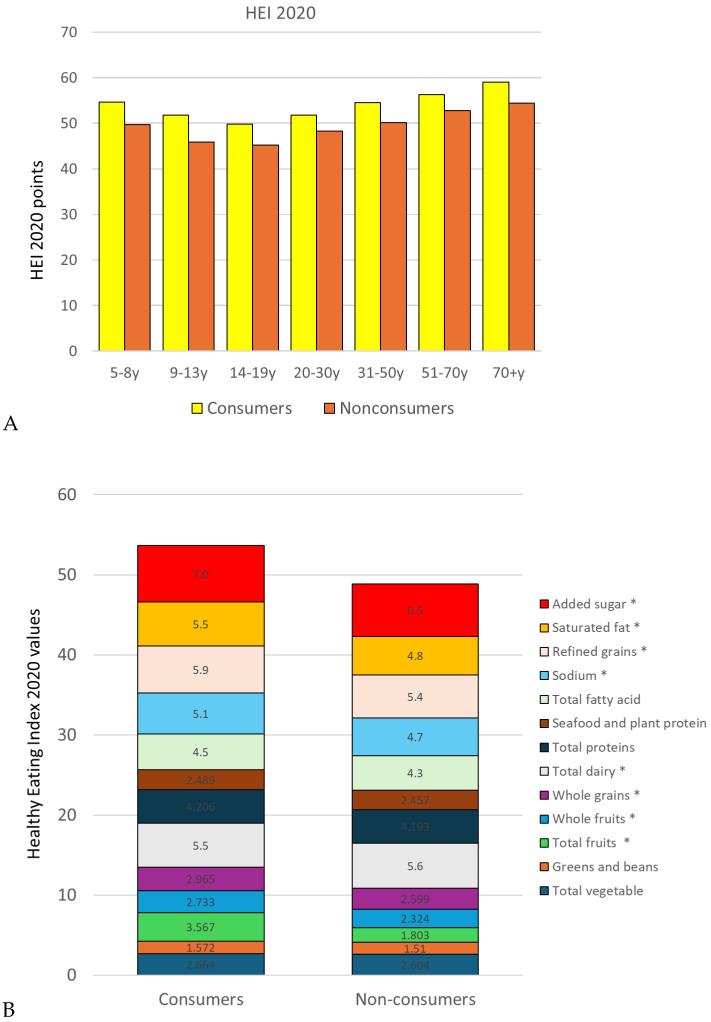
Total HEI 2020 scores (**A**) and HEI 2020 sub scores (**B**) by age group among consumers and non-consumers of 100% fruit juices and diluted juices. Values are means adjusted for total energy intake, age, gender, ethnicity, and IPR. Asterisks represent a significant difference (*p* < 0.05) between consumers and non-consumers of 100% fruit juices (+diluted) after adjustment based on total energy intake, age group, gender, ethnicity, and IPR.

The HEI 2020 is a measure of compliance with Dietary Guidelines. As shown in Figure 5A, higher HEI 2020 dietary quality scores were obtained for consumers of 100% fruit juices at all ages (*p* < 0.001 with or without adjustment). Adjusted mean values were 53 for consumers and 48 for non-consumers, and raw mean values are available in Supplementary Table S4.

After adjustment, Figure 5B shows that consumers of 100% juice and diluted 100% juice had significantly higher HEI 2020 sub scores for selected categories, notably total fruit, whole fruit, and whole grains. More favorable mean HEI 2020 sub scores were observed for refined grains, sodium, saturated fats, and added sugars. There were no significant differences for the other categories, all after adjustment based on energy intake and socio-demographics, except total dairy for which non-consumers had a more favorable sub score.

**Figure 6:**
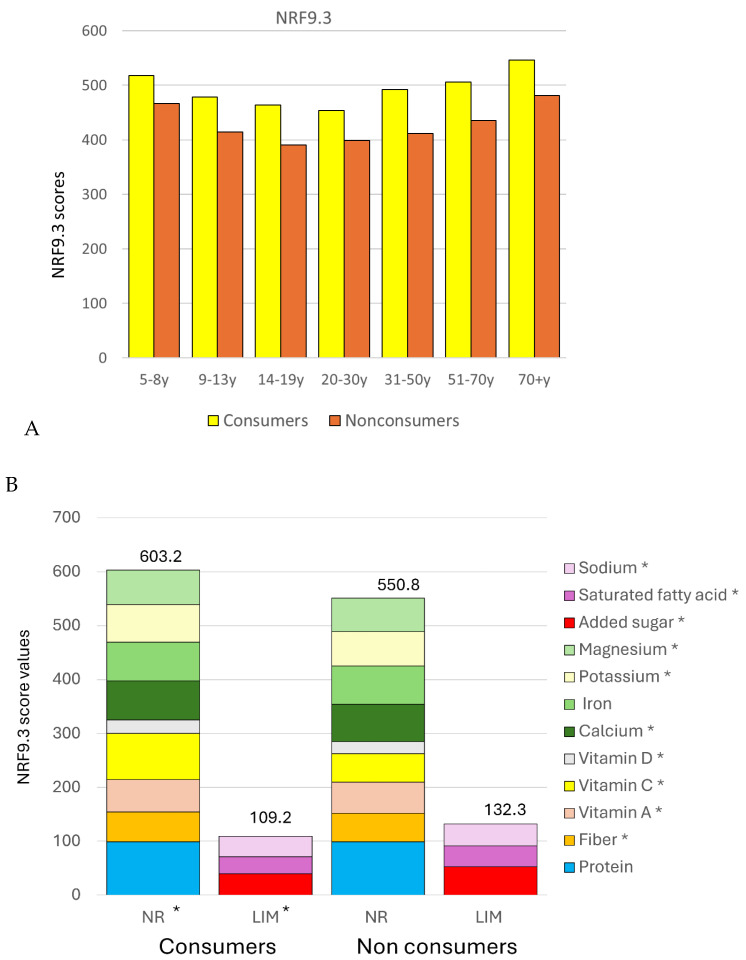
Total NRF 9.3 scores (**A**) and sub scores (**B**) by age group among consumers and non-consumers of 100% fruit juices and diluted 100% juices. Values are means adjusted for total energy intake, age, gender, ethnicity, and IPR. Asterisks represent a significant difference (*p* < 0.05) between consumers and non-consumers of 100% fruit juices after adjustment based on total energy intake, age group, gender, ethnicity, and IPR.

The NRF9.3 score is a calorie-adjusted measure of dietary nutrient density. Figure 6A shows that consumers had higher NRF9.3 scores than non-consumers (*p* < 0.001 with or without adjustment for energy intake and socio-demographics). After adjustment, higher NRF sub scores were obtained for all nutrients to encourage (except proteins and iron), and lower NRF sub scores were obtained for all nutrients to limit. Supplementary Table S5 shows unadjusted means of NRF scores and sub scores between consumers and non-consumers of 100% fruit juices.

## 4. Discussion

### 4.1. Low Consumption of 100% Fruit Juice

The consumption of 100% juice was generally low, both in terms of consumer prevalence and the amounts consumed. Only 24% of the NHANES sample consumed any 100% juice over the 2 days of NHANES dietary recalls. Up to 75% of consumers in the 14–70 y range consumed no 100% juice at all. The median consumption (=50th percentile) of 100% juice was zero. The consumption of diluted 100% juice was practically nonexistent. Water, milk, and sweetened beverages, both regular and diet, were consumed in much higher amounts.

Consumption percentages and the amounts consumed were higher for younger children and for older adults. There was also a strong social gradient in 100% juice consumption and significant effects of incomes and race/ethnicity. Lower incomes were associated with lower consumption of whole fruit and higher consumption of 100% fruit juice. Consistent with past observations, also based on representative NHANES data, lower income groups drank more 100% juice and more milk. Higher-income groups drank more water (bottled and tap) and more low-calorie beverages. The socioeconomic gradients in 100% juice as opposed to whole fruit consumption have been observed before [20].

### 4.2. Meeting Recommendations for 100% Juice

The DGA recommendations for 100% juice consumption were readily met. The DGA recommendations for children (1–6 y) are to limit 100% juice consumption to no more than 4–6 oz/day (^1^/_2_ to ^3^/_4_ cups). The recommendation of older children (7–18 y) is to limit 100% juice to no more than 8 oz/day (1 cup). While there is no strict limit for adults, the DGA recommends that 100% juice should be moderated and not displace whole fruits.

The current consumption patterns did not reach that level. The 1 cup limit (8 oz) was met by the overwhelming majority (94.8%) of the population. The ^1^/_2_. cup limit (4 oz) was met by 88% of the population. Most young children (77%) consumed less than ^1^/_2_ cup of 100% juice per day. Practically all older children (94%) consumed less than 1 cup of 100% juice per day. The percentage of individuals consuming more than 2 cups/day of 100% juice varied by IPR and race/ethnicity. The consumption of 100% juice was higher among lower income and some minority groups.

There was no evidence that 100% juice was displacing whole fruit on a population basis. The DGA recommends that at least 50% of total fruit come from whole fruit rather than 100% juice. The present analyses of whole fruit consumers showed that the average ratio of whole fruit/total fruit was 0.6 in favor of whole fruit. Populations where 100% fruit juices accounted for >50% of total fruit intake were younger children, lower income groups, and minorities—notably non-Hispanic Black children. This was most likely due to the higher cost of whole fruit. Analyses of the USDA national food prices (used in creation of the 2021 Thrifty Food Plan) indicate that 100% fruit juices cost less per serving that do whole fruit. Fruit, 100% juice, and vegetables are important components of diets for mothers, infants, and children [21].

It should be noted that two federal agencies take what might be construed as opposing positions regarding 100% fruit juice. Based on published information, 100% fruit juice meets the proposed definition of “healthy” as developed by the Food and Drug Administration [22]. The 100% juice does contain 1 cup of fruit per RACC and contains zero added sugar. Based on the proposed front-of-pack labeling scheme, recently proposed by the FDA, 100% fruit juice will be low in added sugar, sodium, and saturated fat, with less than 5% of the daily value per serving [23]. That places the USDA in the position of proposing limits on the consumption of a beverage that another federal agency has classified as “healthy”.

Existing guidelines on 100% juice consumption from outside the US are mostly for infants and children from birth to 24 months. That population was not included in the present study. The World Health Organization recommends limiting the consumption of 100% juice by infants and young children under 24 months of age, albeit based on conditional, low certainty evidence [24]. The Pan American Health Organization recommends limiting the amount of juice to avoid displacing more nutrient-rich beverages [25]. Canada, Australia, and new Zealand do not recommend fruit juices for infants and toddlers [25]. It is worth noting that the DGA and international bodies recommend drinking plain water as the beverage of choice. In the present analyses, drinking water was the most popular beverage in the US diet and was consumed in the greatest amounts by far.

### 4.3. 100% Juice Consumption and Diet Quality Metrics

Past analyses of NHANES datasets showed that beverage patterns that featured milk and 100% fruit juices were associated with higher quality diets. That observation supports results previously obtained in studies conducted in the US and elsewhere [26,27,28,29,30]. The present analyses used two metrics of diet quality: the Healthy Eating Index HEI 2020, which tracks compliance with the DGA, and the diet level Nutrient Rich Food (NRF9.3) score.

The consumption of 100% juice was associated with higher HEI 2020 total scores and selected sub scores. Diets of 100% juice consumers were higher not only in total fruit but also in total vegetables, greens and beans, total fruits, whole fruits, whole grains, dairy, protein, and seafood and plant proteins. Significantly, diets of 100% juice consumers were lower in added sugar, saturated fat, and sodium. That would suggest that 100% fruit juice consumption is a marker or an indicator of healthier diets. Higher HEI 2020 scores are indicative of better adherence to the DGA.

The NRF9.3 dietary nutrient density score provided higher values for 100% juice consumers. Their diets were higher in vitamin C and had more potassium. The diet was higher in total sugar but lower in added sugar. The LIM sub score was more favorable for 100% juice consumers. Their diets were lower in sodium, saturated fat, and added sugar. It is likely the 100% juice does not replace whole fruit but may replace sugar-sweetened beverages, the major source of dietary added sugars in the US. In other words, positioning fruit juice versus whole fruit may be misguided. Water, milk, and 100% juice are potential alternatives to the far more prevalent sugar-sweetened beverages.

### 4.4. Limitations

The study had limitations. The NHANES 24 h dietary recalls are self-reports that are subject to misreporting and recall bias. Reports for younger children are obtained from parents or caregivers. Two days of recalls are not necessarily indicative of a habitual diet. The cross-sectional study design of NHANES precludes any considerations of causality; any links to health variables need to be viewed only as associations. Residual confounding may involve physical activity, lifestyles, or other unmeasured variables.

## 5. Conclusions and Policy Recommendations

The DGA recommendations regarding 100% juice were more readily met by the US non-Hispanic White and wealthier populations and less readily by children of minority groups. As noted previously on several occasions, compliance with the USDA dietary guidelines often involves substantially higher costs. The strong relation between whole fruit consumption and higher incomes points to the difficulties that people have in adhering to dietary guidelines. Compliance with dietary guidelines does have an economic cost that is not always adequately acknowledged. The issue of economic access to healthy foods needs to be discussed by the future DGA.

## Figures and Tables

**Table 1:** Beverage categorization.

Beverage Category	Classification Criteria Based on WWEAI Categories
100% Fruit juice	100% juices w/o added sugars: citrus juice; other fruit juice; apple juice; vegetable juice; baby juice, smoothie w/o added sugar.
100% diluted fruit juice	Diluted 100% juices with a fruit juice cup equivalent > 0 and without added sugar: fruit drinks.
Milk and flavored milk	Milk, low fat; flavored milk, low-fat; milk, reduced fat; flavored milk, reduced fat; milk, whole; flavored milk, whole; milk, nonfat; flavored milk, nonfat
Drinking water	Tap water; bottled water; baby water.
Regular sugar-sweetened beverages (SSBs)	Beverages with ≥50 kcal/240 g of beverage (1 cup): coffee, tea, soft drinks, milk shakes and other dairy drinks, diet sport and energy drinks, other diet drinks, diet soft drinks, milk substitutes, fruit drinks, smoothies and grain drinks, sport and energy drinks, nutritional beverages, flavored or carbonated water, enhanced water, fruit drinks (with added sugars), other fruit juice (with added sugars), citrus juice (with added sugars).
Low-calorie beverages (LCBs)	Beverages with ≤50 kcal/240 g of beverage (1 cup)

**Table 2:** Reference daily values (RDVs) for nutrients to encourage and nutrients to limit used in the NRF9.3, based on a 2000 kcal diet.

Nutrient	Reference Value
Protein	50 g/d
Fiber	28 g/d
Vitamin A	900 mcg RAE/d
Vitamin C	90 mg/d
Iron	18 mg/d
Potassium	3500 mg/d
Magnesium	420 mg/d
Calcium	1300 mg/d
Vitamin D	20 mcg/d
Added sugar	50 g/d
Saturated fatty acid	20 g/d
Sodium	2300 mg/d

**Table 3:** Percentages of NHANES participants by age group and socio-demographic variables ^1^.

			Children (n = 4086)				Adults (n = 10,925)				
Percentages		All	5–19 y	5–8 y	9–13 y	14–19 y	20–70 y	20–30 y	31–50 y	51–70 y	>70 y
**All** (% of total)		100	20.6	5.2	7.3	8.1	79.4	15.2	26.9	27.2	10.1
**Gender**	Male	48.7	51.0	52.1	49.7	51.3	48.1	50.5	49.3	47.3	43.6
	Female	51.3	49.0	47.9	50.3	48.7	51.9	49.5	50.7	52.7	56.4
**IPR**	<1	13.0	19.0	21.1	16.9	19.6	11.5	16.6	12.0	10.3	5.4
	1–1.99	17.1	20.5	22.4	20.2	19.7	16.2	17.7	16.0	14.3	19.8
	2–3.49	20.4	20.3	19.2	22.5	19.1	20.4	23.0	19.6	17.6	26.0
	≥3.5	39.0	29.9	28.3	31.0	29.8	41.3	30.5	42.8	47.7	36.6
	NA	10.5	10.2	9.0	9.3	11.8	10.6	12.2	9.6	10.1	12.1
**Ethnicity**	Other Hispanic	8.8	9.9	11.2	9.3	9.6	8.5	12.0	9.3	7.1	4.8
	Other race	10.7	14.0	13.2	14.5	14.1	9.9	11.8	11.6	8.8	5.6
	NH black	11.7	12.7	12.1	12.3	13.4	11.5	13.6	11.7	11.4	7.9
	NH white	59.5	48.1	48.3	50.4	45.8	62.5	52.1	56.8	67.7	79.4
	Mex American	9.2	15.3	15.2	13.5	17.1	7.7	10.5	10.7	5.0	2.3

^1^ *p* values of Chi-square tests by age group: <0.05 (*p* = 0.029 for gender, <0.001 for IPR, and <0.001 for ethnicity).

**Table 4:** Percentage of consumers/non-consumers of 100% fruit juices by socio-demographic variables (n = 15,011).

Variable		Consumers (n = 4110)	Non-Consumers (n = 10,901)	*p* ^1^
**All**		24.5	75.5	
**Age group**	5–8 y	45.4	54.6	<0.001
	9–13 y	37.5	62.5	
	14–19 y	23.7	76.3	
	20–30 y	19.2	80.8	
	31–50 y	19.4	80.6	
	51–70 y	22.9	77.1	
	>70 y	30.8	69.2	
**Ethnicity**	Other Hispanic	31.6	68.4	<0.001
	Other race	22.4	77.6	
	Non-Hispanic White	21.3	78.7	
	Non-Hispanic Black	34.4	65.6	
	Mexican American	28.0	72.0	
**Gender**	Male	25.1	74.9	0.361
	Female	23.9	76.1	
**IPR**	<1	29.6	70.4	0.001
	1–1.99	26.6	73.4	
	2–3.49	24.0	76.0	
	≥3.5	22.3	77.7	
	NA	23.6	76.4	

^1^ *p* values of Chi-square test between consumers of 100% fruit juices status and socio demographical variables.

**Table 5:** Distribution of servings of 100% fruit juices by age groups (n = 15011) ^1^.

	Number of Servings in Cups/Day				
**Age**	**<0.5**	**0.5 to 1.0**	**1.0 to 1.5**	**1.5 to 2.0**	**≥2**
	**%**	**%**	**%**	**%**	**%**
All	87.1	7.7	3.1	0.9	1.2
5–8 y	77.0	14.9	4.6	1.8	1.8
9–13 y	82.0	11.2	5.2	0.9	0.6
14–19 y	86.6	6.7	4.4	0.9	1.3
20–30 y	89.7	5.4	2.3	0.8	1.8
31–50 y	88.5	6.9	2.8	0.6	1.1
51–70 y	88.2	7.0	2.7	1.1	1.0
>70 y	85.5	9.8	3.2	0.6	0.9

^1^ Chi-square test was significant (*p* < 0.001).

## Data Availability

Publicly available datasets are located at: https://wwwn.cdc.gov/nchs/nhanes/search/datapage.aspx?Component=Dietary&Cycle=2017-2020 (accessed on 17 July 2025) and at https://wwwn.cdc.gov/nchs/nhanes/search/datapage.aspx?Component=Dietary&Cycle=2021-2023(accessed on 17 July 2025). The original contributions presented in the study are included in the article/Supplementary Materials, further inquiries can be directed to the corresponding author.

## Supplementary Materials

The following supporting information can be downloaded at: https://www.mdpi.com/article/10.3390/nu17162715/s1, Supplementary Table S1: Distribution of beverage consumption (g/d) by age group (n = 15,011); Supplementary Table S2: Distribution of beverage consumption (in g/d) among children by socio-demographic variables (n = 4086); Supplementary Table S3: Distribution of beverage consumption among adults by socio-demographic variables (n = 10,925); Supplementary Table S4: Percentage of population by number of servings of 100% fruit juices (+diluted), by socio demographic variables among children and adults; Supplementary Table S5. Score of dietary quality among consumers and non-consumers of 100% fruit juices (+diluted).
