# Supplementary material for: Consumption of 100% Juice and Diluted 100% Juice Is Associated with Better Compliance with Dietary Guidelines for Americans: Analyses of NHANES 2017–2023
Source: Nutrients. 2025 Aug 21;17(16):2715. doi: 10.3390/nu17162715 (PMC12389055; doi:10.3390/nu17162715)
Supplement: Supplementary file 1 [file nutrients-17-02715-s001.zip › nutrients-3818125-supplementary.pdf]

Supplementary tables

Supplementary table S1. Distribution of beverage consumption (g/d) by age group (n = 15 011)

Beverage	Age	Mean	Std	95% LCL	95% UCL	P10	P25	P50	P75	P90	Pvalue
**100% + Diluted Juices**	**All**	46.8	113.9	43.2	50.4	0	0	0	0	178.2	0.05
	5-8 y	81.8	130.7	70.8	92.8	0	0	0	124	248	
	9-13 y	63.3	107.2	53.4	73.1	0	0	0	104.5	207.7	
	14-19 y	49.1	118.8	38.4	59.7	0	0	0	0	186	
	20-30 y	40.8	113.7	30.4	51.3	0	0	0	0	155	
	31-50 y	40.1	114.2	34.6	45.6	0	0	0	0	157.5	
	51-70 y	43.0	106.8	39.3	46.7	0	0	0	0	155	
	>70 y	52.2	118.6	43	61.4	0	0	0	65.1	178.2	
**100% Fruit juices**	**All**	45.6	112.1	42	49.2	0	0	0	0	170.5	**<0.001**
	5-8 y	78.3	129.2	67	89.7	0	0	0	124	232.5	
	9-13 y	60.7	104.3	50.9	70.4	0	0	0	104.5	186	
	14-19 y	47.5	117.5	36.9	58	0	0	0	0	178.2	
	20-30 y	40.5	113.1	29.8	51.1	0	0	0	0	155	
	31-50 y	39.2	111.7	33.7	44.6	0	0	0	0	155	
	51-70 y	41.8	104.7	38.3	45.4	0	0	0	0	155	
	>70 y	51.2	118.5	42.1	60.3	0	0	0	62	178.2	
**100% diluted fruit juice**	**All**	1.2	15.3	0.9	1.6	0	0	0	0	0	**<0.001**
	5-8 y	3.5	27.3	1	6	0	0	0	0	0	
	9-13 y	2.6	21.5	0.8	4.5	0	0	0	0	0	
	14-19 y	1.6	20.5	0.4	2.8	0	0	0	0	0	
	20-30 y	0.4	7.3	-0.1	0.8	0	0	0	0	0	
	31-50 y	0.9	14.3	0.4	1.4	0	0	0	0	0	
	51-70 y	1.2	13.9	0.3	2	0	0	0	0	0	
	>70 y	1.0	10.4	-0.2	2.1	0	0	0	0	0	
**Milk and flavored milk**	**All**	101.5	176	96	107	0	0	0	147.9	320.3	**<0.001**
	5-8 y	220.9	207.1	199.9	242	0	61	183	320.3	488	
	9-13 y	185.8	193.5	172	199.6	0	0	137.2	285	429.6	
	14-19 y	130.9	214.6	115.4	146.4	0	0	0	201.5	360.9	
	20-30 y	72.4	159.9	63.7	81.1	0	0	0	79.2	264.3	
	31-50 y	74.3	152.7	64.3	84.3	0	0	0	106.8	248	
	51-70 y	85.6	165.9	76.7	94.5	0	0	0	122	284.7	
	>70 y	114	169.1	101.2	126.8	0	0	30.5	167.8	343.1	
**Drinking water, tap and bottled**	**All**	1201.4	1132.1	1162.3	1240.6	120	457.5	975	1663.5	2454	**<0.001**
	5-8 y	567.4	453.9	526.6	608.3	75	250	480	769.7	1140	
	9-13 y	699.6	557.1	659.4	739.9	120	352.5	600	967.2	1305	
	14-19 y	1159.8	983.3	1072.9	1246.6	172.5	480	960	1614	2353.5	
	20-30 y	1437.5	1153.4	1343.9	1531.1	180	613.5	1219.5	2006.2	3042	
	31-50 y	1521.5	1480.9	1428.9	1614.1	240	690	1314.8	2010	2934	
	51-70 y	1146.9	936.5	1102.2	1191.6	97.5	435	987	1620	2319	
	>70 y	861.8	711.5	828.8	894.7	90	330	720	1231.5	1792.5	
**SSB**	**All**	312.7	443.8	295	330.4	0	0	183	449.5	813.9	**<0.001**
	5-8 y	164.4	228.2	144.5	184.3	0	0	93	248	433	
	9-13 y	280.5	275.5	255.7	305.2	0	62	216	426.2	627.8	
	14-19 y	352.3	368.8	327.6	377	0	62	269.9	510	802	
	20-30 y	400.9	441.6	367.5	434.4	0	0	286.8	616	970	
	31-50 y	380	545.9	343.4	416.6	0	0	192	513	1054	

Beverage	Age	Mean	Std	95% LCL	95% UCL	P10	P25	P50	P75	P90	Pvalue
	51-70 y	276.2	449.5	249.7	302.7	0	0	100.8	386	794.2	
	>70 y	166.6	256.3	152.3	180.9	0	0	0	256	528	
**LCB**	**All**	445.4	593.3	421.5	469.3	0	0	270	636.2	1095	**<0.001**
	5-8 y	45.3	124.9	33.9	56.7	0	0	0	0	150	
	9-13 y	57.7	126.9	48	67.4	0	0	0	45	221.5	
	14-19 y	106.4	214	92.2	120.5	0	0	0	157.5	325.5	
	20-30 y	272.2	414.7	239.5	304.9	0	0	147.4	378.1	690	
	31-50 y	515.8	650.4	470.5	561.1	0	97.5	360	720	1140	
	51-70 y	695.8	682.4	655.8	735.8	0	240	540	950.3	1530	
	>70 y	604.6	505.2	577	632.2	90	255	510.6	800.5	1200	

Supplementary table S2. Distribution of beverage consumption (in g/d) among Children by socio-demographical variables (n = 4 086)

Beverage	Variable		Mean	Std	95% LCL	95% UCL	P10	P25	P50	P75	P90	P.value
**100% + Diluted Fruit juices**	**All**		62.4	118.7	56.3	68.5	0.0	0.0	0.0	100.8	216.0	
	**Ethnicity**	Other hispanic	94.1	149.2	67.4	120.8	0.0	0.0	0.0	139.5	310.0	0.427
		Other race	55.7	126.6	41.5	70.0	0.0	0.0	0.0	62.0	186.0	
		Non hispanic black	84.4	124.7	73.2	95.7	0.0	0.0	0.0	124.0	262.0	
		Non hispanic white	52.0	108.8	44.2	59.8	0.0	0.0	0.0	62.0	186.0	
		Mexican American	62.5	107.8	52.6	72.4	0.0	0.0	0.0	102.7	201.5	
	**Gender**	Male	66.8	126.8	58.4	75.2	0.0	0.0	0.0	104.5	228.5	0.061
		Female	57.8	109.6	51.2	64.4	0.0	0.0	0.0	93.0	207.7	
	**IPR**	<1	75.0	127.2	62.1	87.9	0.0	0.0	0.0	124.0	248.0	0.395
		1–1.99	63.9	113.5	50.2	77.6	0.0	0.0	0.0	104.5	209.0	
		2–3.49	58.9	120.1	44.8	72.9	0.0	0.0	0.0	62.0	205.2	
		≥3.5	60.0	120.6	49.4	70.6	0.0	0.0	0.0	93.0	213.0	
		NA	50.1	102.0	36.3	63.9	0.0	0.0	0.0	62.0	186.0	
**100% fruit juices**	**All**		60.0	116.8	53.9	66.0	0.0	0.0	0.0	93.0	201.5	
	**Ethnicity**	Other hispanic	92.1	148.0	65.7	118.4	0.0	0.0	0.0	131.8	294.5	0.455
		Other race	54.8	126.2	40.4	69.3	0.0	0.0	0.0	62.0	186.0	
		Non hispanic white	48.2	105.1	40.9	55.6	0.0	0.0	0.0	54.2	178.2	
		Non hispanic black	83.2	124.3	72.0	94.4	0.0	0.0	0.0	124.0	248.0	
		Mexican American	61.4	107.0	51.8	70.9	0.0	0.0	0.0	100.8	193.7	
	**Gender**	Male	64.1	125.1	55.7	72.5	0.0	0.0	0.0	100.8	209.2	0.067
		Female	55.6	107.3	49.1	62.1	0.0	0.0	0.0	77.5	186.0	
	**IPR**	<1	73.6	126.1	60.8	86.4	0.0	0.0	0.0	124.0	248.0	0.258
		1–1.99	63.5	113.2	49.9	77.2	0.0	0.0	0.0	104.5	209.0	
		2–3.49	55.8	116.6	42.4	69.1	0.0	0.0	0.0	62.0	186.0	
		≥3.5	55.5	117.6	45.0	66.1	0.0	0.0	0.0	77.5	186.0	
		NA	48.6	101.1	34.7	62.5	0.0	0.0	0.0	62.0	174.0	

Beverage	Variable		Mean	Std	95% LCL	95% UCL	P10	P25	P50	P75	P90	P.value
**100% diluted fruit juice**	**All**		2.4	22.8	1.4	3.5	0.0	0.0	0.0	0.0	0.0	
	**Ethnicity**	Other hispanic	2.0	24.8	-0.2	4.2	0.0	0.0	0.0	0.0	0.0	0.646
		Other race	0.9	12.4	0.0	1.8	0.0	0.0	0.0	0.0	0.0	
		Non hispanic white	3.7	28.2	1.7	5.7	0.0	0.0	0.0	0.0	0.0	
		Non hispanic black	1.2	12.0	0.3	2.2	0.0	0.0	0.0	0.0	0.0	
		Mexican American	1.1	15.0	0.0	2.2	0.0	0.0	0.0	0.0	0.0	
	**Gender**	Male	2.7	22.6	1.0	4.3	0.0	0.0	0.0	0.0	0.0	0.624
		Female	2.2	23.0	1.2	3.2	0.0	0.0	0.0	0.0	0.0	
	**IPR**	<1	1.4	20.4	0.2	2.6	0.0	0.0	0.0	0.0	0.0	**0.004**
		1–1.99	0.4	8.1	0.0	0.7	0.0	0.0	0.0	0.0	0.0	
		2–3.49	3.1	24.1	0.3	5.9	0.0	0.0	0.0	0.0	0.0	
		≥3.5	4.5	30.2	1.6	7.3	0.0	0.0	0.0	0.0	0.0	
		NA	1.5	18.2	-0.3	3.3	0.0	0.0	0.0	0.0	0.0	
**Milk and flavored milk**	**All**		173.1	208.6	161.9	184.4	0.0	0.0	124.0	269.4	429.0	
	**Ethnicity**	Other hispanic	169.9	229.4	143.9	195.9	0.0	0.0	124.0	244.0	383.8	**0.019**
		Other race	163.5	192.1	143.5	183.6	0.0	0.0	122.0	259.3	388.3	
		Non hispanic white	189.3	225.9	173.4	205.3	0.0	0.0	124.0	301.4	468.7	
		Non hispanic black	111.6	142.7	100.4	122.8	0.0	0.0	61.0	180.5	309.0	
		Mexican American	184.2	188.4	163.8	204.5	0.0	0.0	152.5	286.7	442.3	
	**Gender**	Male	203.2	226.4	185.9	220.5	0.0	0.0	152.5	308.0	463.7	**<0.001**
		Female	141.9	183.3	130.4	153.4	0.0	0.0	86.4	228.8	368.0	
	**IPR**	<1	185.9	191.5	166.8	204.9	0.0	0.0	147.4	294.5	431.0	**<0.001**
		1–1.99	207.0	210.8	190.4	223.6	0.0	0.0	172.8	307.6	495.6	
		2–3.49	149.8	191.2	127.4	172.2	0.0	0.0	91.5	233.8	393.4	
		≥3.5	159.2	214.3	134.9	183.4	0.0	0.0	106.8	246.5	425.0	
		NA	168.7	239.9	131.2	206.3	0.0	0.0	108.5	248.0	412.5	
**Drinking water, tap**	**All**		847.1	779.7	799.5	894.7	120.0	355.0	660.0	1117.5	1792.5	

Beverage	Variable		Mean	Std	95% LCL	95% UCL	P10	P25	P50	P75	P90	P.value
**and bottled**												
	**Ethnicity**	Other hispanic	810.8	726.7	692.8	928.8	102.0	300.0	622.5	1057.5	1845.0	**0.018**
		Other race	856.1	735.0	780.9	931.2	180.0	405.0	690.0	1080.0	1720.5	
		Non hispanic white	885.5	810.5	800.2	970.7	126.8	367.5	682.5	1117.5	1800.0	
		Non hispanic black	604.6	570.6	560.7	648.4	0.0	190.1	463.5	867.0	1240.5	
		Mexican American	942.7	861.3	822.8	1062.6	121.5	373.5	697.5	1297.5	1947.0	
	**Gender**	Male	848.9	842.8	796.5	901.4	105.0	330.0	627.2	1080.0	1872.0	0.911
		Female	845.2	708.4	782.3	908.1	126.8	360.0	683.6	1120.5	1725.0	
	**IPR**	<1	739.4	728.8	682.6	796.1	52.5	253.5	517.5	1014.0	1701.0	**0.005**
		1–1.99	763.4	746.7	693.4	833.5	52.5	255.0	570.0	1035.0	1641.8	
		2–3.49	893.7	798.9	791.9	995.4	126.8	375.0	690.0	1157.5	1882.5	
		≥3.5	947.9	782.8	846.7	1049.1	232.5	472.5	760.5	1202.5	1800.0	
		NA	828.6	844.9	677.9	979.3	102.0	277.5	577.5	1080.0	1800.0	
**SSB**	**All**		279.2	314.5	263.1	295.4	0.0	0.0	193.8	418.7	657.0	
	**Ethnicity**	Other hispanic	240.7	263.2	212.2	269.3	0.0	0.0	186.0	356.5	555.0	0.237
		Other race	235.5	284.4	206.2	264.7	0.0	0.0	162.8	356.5	620.0	
		Non hispanic white	299.0	347.6	276.4	321.5	0.0	0.0	201.2	446.2	728.2	
		Non hispanic black	284.7	292.9	263.6	305.8	0.0	46.0	201.2	434.0	658.8	
		Mexican American	277.6	270.6	246.5	308.8	0.0	46.0	240.2	414.0	634.2	
	**Gender**	Male	309.3	342.4	287.1	331.5	0.0	0.0	224.8	459.8	744.0	**<0.001**
		Female	248.0	279.3	229.8	266.3	0.0	0.0	186.0	376.8	573.5	
	**IPR**	<1	310.5	336.1	279.3	341.8	0.0	57.5	244.5	434.0	740.5	0.173
		1–1.99	281.3	294.3	254.5	308.1	0.0	23.2	201.2	403.0	651.2	
		2–3.49	285.8	351.1	253.6	318.1	0.0	0.0	186.0	435.0	682.0	
		≥3.5	262.5	290.2	229.9	295.0	0.0	0.0	189.0	426.2	605.5	
		NA	252.9	298.8	203.6	302.2	0.0	0.0	186.0	372.0	608.0	
**LCB**	**All**		73.7	168.4	65.4	82.0	0.0	0.0	0.0	75.0	253.5	
	**Ethnicity**	Other hispanic	58.1	124.4	42.5	73.7	0.0	0.0	0.0	75.0	195.0	0.943
		Other race	67.3	176.2	49.3	85.4	0.0	0.0	0.0	17.2	240.0	
		Non hispanic white	89.1	187.4	74.9	103.4	0.0	0.0	0.0	120.0	292.5	
		Non hispanic black	48.8	121.0	40.0	57.6	0.0	0.0	0.0	0.0	180.0	
		Mexican American	61.8	151.3	45.8	77.7	0.0	0.0	0.0	54.2	217.5	

Beverage	Variable		Mean	Std	95% LCL	95% UCL	P10	P25	P50	P75	P90	P.value
	**Gender**	Male	78.9	185.6	65.3	92.5	0.0	0.0	0.0	75.0	259.3	0.221
		Female	68.3	148.2	59.0	77.6	0.0	0.0	0.0	75.0	248.0	
	**IPR**	<1	60.7	153.4	46.7	74.7	0.0	0.0	0.0	46.0	190.1	0.069
		1–1.99	61.7	145.3	52.5	70.8	0.0	0.0	0.0	60.0	217.5	
		2–3.49	80.2	170.7	63.0	97.5	0.0	0.0	0.0	90.0	300.0	
		≥3.5	86.9	186.5	69.1	104.8	0.0	0.0	0.0	104.6	270.0	
		NA	70.6	175.2	45.1	96.1	0.0	0.0	0.0	61.0	240.0	

Supplementary table S3. Distribution of beverage consumption among Adults by socio-demographical variables (n = 10 925)

Beverage	Variable		Mean	Std	95% LCL	95% UCL	P10	P25	P50	P75	P90	P.value
**100% + Diluted Juices**	**All**		42.8	112.3	39.1	46.4	0	0	0	0	162.8	
	**Ethnicity**	Other hispanic	61.3	135.9	49.6	73	0	0	0	62	224.8	0.98
		Other race	36.3	98.1	29.2	43.3	0	0	0	0	124	
		Non hispanic black	69.3	152.6	61	77.5	0	0	0	93	248	
		Non hispanic white	36.2	99.9	31.1	41.3	0	0	0	0	131.8	
		Mexican American	44.2	116.6	34.3	54.1	0	0	0	0	162.8	
	**Gender**	Male	47.2	119	42.2	52.3	0	0	0	0	186	**0.008**
		Female	38.6	105.5	34.2	43	0	0	0	0	139.5	
	**IPR**	<1	55.7	138.7	45.8	65.7	0	0	0	0	201.5	**0.001**
		1–1.99	49.8	131.1	40.9	58.8	0	0	0	0	185.8	
		2–3.49	43.6	111.8	35.4	51.8	0	0	0	0	162.8	
		≥3.5	36	93.5	31.4	40.6	0	0	0	0	131.8	
		NA	42.5	115.3	35.1	49.9	0	0	0	0	155	
**100% Fruit juices**	**All**		41.9	110.6	38.2	45.5	0	0	0	0	157.2	
	**Ethnicity**	Other hispanic	59.9	134.4	48.2	71.5	0	0	0	62	224.8	0.999
		Other race	36	97.8	29	43	0	0	0	0	124	
		Non hispanic white	35.2	97.7	30.2	40.3	0	0	0	0	124	
		Non hispanic black	68.3	150.9	59.8	76.7	0	0	0	93	248	
		Mexican American	44.1	116.5	34.2	53.9	0	0	0	0	162.8	
	**Gender**	Male	46.5	116.7	41.5	51.5	0	0	0	0	186	**0.005**
		Female	37.6	104.4	33.3	41.9	0	0	0	0	124	
	**IPR**	<1	54.7	137	45.1	64.3	0	0	0	0	201.5	**0.001**
		1–1.99	49.5	128.8	40.7	58.3	0	0	0	0	178.2	
		2–3.49	42.5	109.6	34.3	50.7	0	0	0	0	155	
		≥3.5	35	92.1	30.4	39.6	0	0	0	0	124	
		NA	41.7	114.3	34.1	49.2	0	0	0	0	155	

Beverage	Variable		Mean	Std	95% LCL	95% UCL	P10	P25	P50	P75	P90	P.value
**100% diluted Fruit juices**	**All**		0.9	12.6	0.6	1.2	0	0	0	0	0	
	**Ethnicity**	Other hispanic	1.4	15.9	0.1	2.8	0	0	0	0	0	0.683
		Other race	0.3	8	-0.2	0.7	0	0	0	0	0	
		Non hispanic white	1	12	0.5	1.5	0	0	0	0	0	
		Non hispanic black	1	18.8	0.4	1.6	0	0	0	0	0	
		Mexican American	0.1	4	-0.1	0.4	0	0	0	0	0	
	**Gender**	Male	0.7	13	0.3	1.2	0	0	0	0	0	0.431
		Female	1	12.3	0.6	1.5	0	0	0	0	0	
	**IPR**	<1	1	16.7	-0.2	2.3	0	0	0	0	0	0.141
		1–1.99	0.4	8.6	0	0.7	0	0	0	0	0	
		2–3.49	1.1	12.6	0.5	1.8	0	0	0	0	0	
		≥3.5	1	12.8	0.5	1.5	0	0	0	0	0	
		NA	0.8	12.1	0.2	1.5	0	0	0	0	0	
**Milk and flavored milk**	**All**		82.8	161.3	76	89.6	0	0	0	114.4	269.4	
	**Ethnicity**	Other hispanic	65	120.4	54.6	75.5	0	0	0	91.5	222.8	0.167
		Other race	75	147.7	64.9	85.1	0	0	0	97.2	259.2	
		Non hispanic white	92.5	175.2	82.9	102.1	0	0	0	122	307.5	
		Non hispanic black	54	121.3	44.8	63.2	0	0	0	45.8	193.2	
		Mexican American	76.5	143.4	64.8	88.3	0	0	0	106.8	259.3	
	**Gender**	Male	96.6	184.3	86.1	107.2	0	0	0	129.6	320.3	**<0.001**
		Female	70	135.3	63.7	76.4	0	0	0	91.5	244	
	**IPR**	<1	87.8	163.3	76	99.6	0	0	0	122	282.1	**0.019**
		1–1.99	86.4	163.8	76.8	96	0	0	0	122	284.7	
		2–3.49	92	176	82	102.1	0	0	0	122	292.3	
		≥3.5	76.1	155.5	67.1	85	0	0	0	98.3	264.3	
		NA	80.6	146.2	67.1	94.1	0	0	0	122	264.3	
**Drinking water, tap**	**All**		1293.6	1189.9	1246.2	1341	126.8	507	1087.5	1777.5	2565	

Beverage	Variable		Mean	Std	95% LCL	95% UCL	P10	P25	P50	P75	P90	P.value
and bottled												
	**Ethnicity**	Other hispanic	1283.5	1024.2	1180.1	1386.9	217.5	555	1080	1720.5	2707.5	0.065
		Other race	1377.9	1080.5	1310.4	1445.5	247.5	652.5	1140	1830	2819.9	
		Non hispanic white	1281.5	1253.8	1218.5	1344.5	120	480	1084.5	1774.5	2535	
		Non hispanic black	1183.4	1020.1	1121.8	1245	120	435	960	1701	2535	
		Mexican American	1459.3	1174.3	1384.5	1534	262.5	667.5	1227	1980	2748	
	**Gender**	Male	1369.2	1397.5	1289.4	1449.1	120	495	1140	1912.5	2900.1	**0.001**
		Female	1223.4	952.8	1187.6	1259.3	171	517.5	1068.8	1740	2400	
	**IPR**	<1	1199.5	1768.8	999.4	1399.6	0	360	885	1620	2391	**0.005**
		1–1.99	1201.1	1090.3	1140.9	1261.2	30	380.2	987	1720.5	2564.6	
		2–3.49	1308.7	1220.3	1195.4	1422	150	502.5	1108.5	1800	2721.7	
		≥3.5	1362.8	1032.2	1310.7	1415	240	660	1200	1860	2617.5	
		NA	1238.2	1045.7	1137.9	1338.4	119.9	480	1032	1725	2527.5	
**SSB**	**All**		321.4	471.3	301.2	341.7	0	0	170	465	875.5	
	**Ethnicity**	Other hispanic	354.3	462	311.3	397.3	0	0	248	516.2	900	0.225
		Other race	302.4	473.6	264.2	340.6	0	0	147.4	434	806	
		Non hispanic white	304.3	488.8	276.6	332.1	0	0	116.2	434	861.4	
		Non hispanic black	375.4	404.1	349.2	401.6	0	20.3	266.9	546.2	920	
		Mexican American	368.5	411.7	327.4	409.6	0	15.5	248	553.5	903	
	**Gender**	Male	391.1	551	363.4	418.8	0	0	209.2	558	1039	**<0.001**
		Female	256.8	371.3	235.5	278.2	0	0	123	379.8	731.8	
	**IPR**	<1	490.4	611.1	440.2	540.7	0	38.8	297.5	682	1302	**<0.001**
		1–1.99	376.5	506	340.9	412.1	0	0	216	542.2	1000.8	
		2–3.49	350.9	512.2	313.6	388.2	0	0	186	482.5	947.1	
		≥3.5	232.4	344.5	212	252.8	0	0	81.3	371	674.2	
		NA	344.9	513.9	296.8	393	0	0	170.5	512	930	
**LCB**	**All**		542.1	625.3	512.9	571.2	0	120	390	742.5	1230	
	**Ethnicity**	Other hispanic	340.6	450.7	299.9	381.4	0	60	240	450.6	743.5	0.408
		Other race	456	578.2	408.2	503.8	0	75	306.8	607.5	1056.2	
		Non hispanic white	660	680.1	625.6	694.4	0	217.5	525	900	1410.2	
		Non hispanic black	236.9	336	209.2	264.7	0	0	120	360	616	
		Mexican American	370.5	411.1	322	419	0	90	266.8	525	840	

Beverage	Variable		Mean	Std	95% LCL	95% UCL	P10	P25	P50	P75	P90	P.value
	**Gender**	Male	578.8	691.3	541.8	615.8	0	105	413	795	1320	**0.001**
		Female	508	555	476.2	539.7	0	120	367.5	706.5	1140	
	**IPR**	<1	367.6	625.5	320.9	414.2	0	0	209.4	480	885	**<0.001**
		1–1.99	455.6	591.7	411.2	500	0	0	285	607.5	1080	
		2–3.49	540.2	658.8	483.2	597.2	0	90	375	748.5	1252.5	
		≥3.5	640.8	623.7	609.7	671.9	0	225	525	856	1387	
		NA	482.1	541.6	423.8	540.4	0	100.8	330	675	1147.5	

Supplementary table S4. Percentage of population by number of servings of 100% fruit juices (+ diluted), by socio demographic variables among children and adults

		All						Children (n = 4 086)						Adults (n = 10 925)					
**Variable**		**<0.5**	**0.5 to 1.0[**	**1.0 to 1.5**	**1.5 to 2.0**	**≥2**	**Pval**	**<0.5**	**0.5 to 1.0**	**1.0 to 1.5**	**1.5 to 2.0**	**≥2**	**Pval**	**<0.5**	**0.5 to 1.0**	**1.0 to 1.5**	**1.5 to 2.0**	**≥2**	**Pval**
**All**		87.1	7.7	3.1	0.9	1.2		82.6	10.4	4.7	1.1	1.2		88.3	7.0	2.7	0.8	1.2	
**Ethnicity**	*Other hispanic*	81.7	9.0	4.8	2.0	2.5	**<0.001**	74.3	12.8	7.6	2.7	2.6	**0.001**	84.0	7.8	3.9	1.8	2.5	**<0.001**
	Other race	89.0	6.5	2.3	0.9	1.3		85.3	10.0	1.6	1.1	2.1		90.3	5.3	2.6	0.8	1.0	
	Non hispanic white	89.0	6.9	2.7	0.5	0.9		85.4	8.3	4.6	0.8	0.9		89.7	6.6	2.3	0.5	0.9	
	Non hispanic black	80.2	11.6	4.3	2.1	1.8		75.2	15.1	6.8	1.7	1.3		81.6	10.5	3.6	2.2	2.0	
	Mexican American	86.5	8.2	4.1	0.6	0.8		82.6	11.8	4.5	0.7	0.4		88.5	6.3	3.8	0.5	1.0	
**Gender**	*Male*	85.4	8.7	3.4	1.0	1.6	**0.001**	81.0	11.5	4.2	1.4	1.8	**0.014**	86.6	7.9	3.2	0.8	1.5	**0.009**
	Female	88.7	6.8	2.9	0.8	0.8		84.1	9.2	5.3	0.8	0.6		89.8	6.2	2.3	0.8	0.9	
**IPR**	*<1*	83.3	9.2	4.4	1.3	1.8	**0.013**	78.3	12.6	6.8	1.6	0.7	0.202	85.4	7.7	3.4	1.2	2.3	0.066
	1–1.99	85.2	8.8	3.3	1.0	1.6		81.9	11.7	4.3	0.6	1.5		86.2	7.9	3.0	1.2	1.7	
	2–3.49	87.6	7.3	2.7	0.9	1.4		83.7	10.2	2.8	1.8	1.5		88.6	6.6	2.7	0.7	1.3	
	≥3.5	88.7	6.8	3.1	0.6	0.7		83.6	9.2	5.3	0.6	1.3		89.7	6.4	2.7	0.6	0.6	
	NA	87.8	8.0	2.3	0.9	1.0		86.5	7.4	4.1	1.5	0.5		88.2	8.1	1.9	0.7	1.1	

Supplementary table S5. Score of dietary quality among consumers and non-consumers of 100% fruit juices (+ diluted)

	**All**				**Consumers (n = 4 110)**				**Non-consumers (n = 10 901)**						
	**Mean**	**Std**	**95% LCL**	**95% UCL**	**Mean**	**Std**	**95% LCL**	**95% UCL**	**Mean**	**Std**	**95% LCL**	**95% UCL**	**Pval1**	**Pval2**	**Pval3**
**NRF9.3 Total score**	453.9	157.7	446.0	461.8	502.9	133.9	496.7	509.2	438.0	161.5	429.1	446.9	**<0.001**	**<0.001**	**<0.001**
**NR**	575.0	115.4	569.8	580.2	608.1	99.8	603.2	612.9	564.3	118.0	558.6	570.0	**<0.001**	**<0.001**	**<0.001**
proteins	99.2	4.3	99.1	99.3	99.2	3.9	99.1	99.4	99.2	4.4	99.0	99.3	0.540	0.163	0.164
fiber	56.1	21.5	55.2	57.1	56.9	20.0	55.9	57.8	55.9	22.0	54.7	57.0	0.120	**0.036**	**0.004**
vitamin A	59.7	26.9	58.5	60.8	60.6	26.0	59.2	62.0	59.3	27.1	58.0	60.6	0.089	**0.044**	**0.013**
vitamin C	63.2	33.7	61.9	64.6	86.9	21.0	85.7	88.1	55.6	33.5	54.2	56.9	**<0.001**	**<0.001**	**<0.001**
vitamin D	22.3	18.0	21.7	22.8	24.4	17.6	23.5	25.2	21.6	18.1	21.0	22.3	**<0.001**	**<0.001**	**<0.001**
calcium	68.5	21.3	67.7	69.3	70.5	20.1	69.5	71.5	67.9	21.6	67.1	68.7	**<0.001**	**<0.001**	**<0.001**
iron	70.5	18.1	69.9	71.0	70.9	17.9	70.2	71.7	70.3	18.1	69.7	70.9	0.168	0.419	0.061
potassium	68.8	17.9	68.1	69.5	72.0	16.7	71.3	72.7	67.8	18.2	66.9	68.6	**<0.001**	**<0.001**	**<0.001**
magnesium	66.8	18.1	66.1	67.5	66.6	16.7	65.9	67.4	66.8	18.5	66.0	67.7	0.729	**0.041**	**0.001**
**LIM**	121.1	71.3	117.5	124.7	105.1	60.0	102.1	108.1	126.3	73.9	122.1	130.4	**<0.001**	**<0.001**	**<0.001**
added sugar	43.0	66.7	40.3	45.8	35.6	52.2	32.9	38.2	45.4	70.7	42.3	48.6	**<0.001**	**<0.001**	**<0.001**
saturated fatty acid	36.3	32.1	34.9	37.7	32.2	29.6	30.5	33.9	37.7	32.7	36.2	39.2	**<0.001**	**<0.001**	**<0.001**
sodium	41.8	36.1	40.6	42.9	37.4	33.0	35.8	39.0	43.2	36.9	41.9	44.4	**<0.001**	**<0.001**	**0.001**
**HEI 2020 Total score**	52.3	13.5	51.5	53.1	55.1	12.7	54.6	55.7	51.4	13.6	50.5	52.3	**<0.001**	**<0.001**	**<0.001**
Total vegetable	2.964	1.551	2.909	3.018	2.88	1.545	2.811	2.949	2.99	1.552	2.923	3.058	**0.023**	0.59	0.233
Greens and beans	1.823	2.1	1.717	1.928	1.776	2.052	1.656	1.896	1.838	2.116	1.72	1.956	0.332	0.436	0.373
Total fruits	2.281	1.959	2.193	2.369	3.572	1.503	3.514	3.63	1.863	1.906	1.767	1.959	**<0.001**	**<0.001**	**<0.001**
Whole fruits	2.519	2.187	2.419	2.618	2.793	2.12	2.688	2.898	2.43	2.201	2.315	2.545	**<0.001**	**<0.001**	**<0.001**
Whole grain	2.676	3.049	2.573	2.779	2.915	3.026	2.763	3.066	2.598	3.053	2.478	2.719	**0.002**	**0.003**	**0.001**
Total dairy	5.192	3.019	5.102	5.281	5.225	2.971	5.075	5.375	5.181	3.035	5.086	5.276	0.577	0.138	**0.048**
Total proteins	4.393	1.051	4.354	4.433	4.337	1.082	4.283	4.392	4.412	1.04	4.366	4.457	**0.021**	0.876	0.684
Seafood and plant proteins	2.822	2.148	2.73	2.913	2.753	2.135	2.649	2.857	2.844	2.152	2.734	2.954	0.186	0.477	0.614
Total fatty acid	4.705	3.368	4.579	4.832	4.659	3.325	4.489	4.828	4.721	3.381	4.578	4.864	0.52	0.327	0.201
Sodium	4.662	3.18	4.55	4.773	5.135	3.095	4.994	5.276	4.508	3.192	4.39	4.627	**<0.001**	**<0.001**	**<0.001**
Refined grains	6.088	3.438	5.981	6.194	6.295	3.301	6.14	6.449	6.02	3.478	5.895	6.146	**0.007**	**<0.001**	**<0.001**
Saturated fat	5.112	3.267	4.97	5.254	5.511	3.172	5.334	5.687	4.983	3.288	4.834	5.132	**<0.001**	**<0.001**	**<0.001**
Added sugar	7.084	3.033	6.957	7.21	7.292	2.705	7.151	7.434	7.016	3.129	6.872	7.16	**0.001**	**<0.001**	**<0.001**

Pval1 : pvalue of the difference between consumers and non-consumers using GLM, without adjustment ; Pval2 : pvalue of the difference between consumers and non-consumers using GLM, with on age (adults vs children/ado), gender, ethnicity, IPR ; Pval3 : pvalue of the difference between consumers and non-consumers using GLM, with on total energy intake, age (adults vs children/ado), gender, ethnicity, IPR
